# Polymorphic Allele of Human *IRGM1* Is Associated with Susceptibility to Tuberculosis in African Americans

**DOI:** 10.1371/journal.pone.0016317

**Published:** 2011-01-21

**Authors:** Katherine Y. King, Justin D. Lew, Ngan P. Ha, Jeffery S. Lin, Xin Ma, Edward A. Graviss, Margaret A. Goodell

**Affiliations:** 1 Department of Pediatrics, Section of Infectious Diseases, Baylor College of Medicine, Houston, Texas, United States of America; 2 Center for Molecular and Translational Human Infectious Diseases Research, The Methodist Hospital Research Institute, Houston, Texas, United States of America; 3 University of California, Berkeley, California, United States of America; 4 Stem Cells and Regenerative Medicine Center, Baylor College of Medicine, Houston, Texas, United States of America; St. Petersburg Pasteur Institute, Russian Federation

## Abstract

An ancestral polymorphic allele of the human autophagy-related gene *IRGM1* is associated with altered gene expression and a genetic risk for Crohn's Disease (CD). We used the single nucleotide polymorphism *rs10065172C*/*T* as a marker of this polymorphic allele and genotyped 370 African American and 177 Caucasian tuberculosis (TB) cases and 180 African American and 110 Caucasian controls. Among African Americans, the TB cases were more likely to carry the CD-related T allele of *rs10065172* (odds ratio of 1.54; 95% confidence interval, 1.17–2.02; *P*<0.01) compared to controls. Our finding suggests that this CD-related *IRGM1* polymorphic allele is also associated with human susceptibility to TB disease among African Americans.

## Introduction

Tuberculosis (TB) disease as a result of *Mycobacterium tuberculosis* infection is the second deadliest infectious disease worldwide, causing 1.8 million deaths annually [1]. Development of active TB disease depends on a complex relationship between the bacterium, environment, and the host. Importantly, only about 10% of presumed immunologically normal individuals who are exposed to *M. tuberculosis* develop disease. Most infected individuals carry the bacteria without overt disease for their entire life, a condition called latent TB infection (LTBI). Among the minority who progress to TB disease, the molecular mechanisms responsible for susceptibility to clinical disease are still poorly understood. Understanding host factors in immunity against the pathogen will inform efforts to prevent and treat TB disease.

The immunity-related GTPase M (IRGM1) is potentially important in the host immune response against TB. This protein is required for immunity against a range of intracellular pathogens in mice, including Listeria, Toxoplasma, and *M. tuberculosis* [2], [3], [4]. However, the role of IRGM1 in humans has been controversial. The gene appears to have a role in autophagy-dependent destruction of *Mycobacterium bovis* (BCG) in cultured human macrophages [5]. An *IRGM1* allele with two single nucleotide polymorphisms (SNPs, *rs13361189* T/C and *rs10065172* C/T) and a 20-kb deletion upstream of the coding region, all in nearly 100% linkage disequilibrium with each other, has been associated with Crohn's disease (CD) [6]. This polymorphic allele is also found to be associated with decreased expression levels in an immortalized lymphoblastoid cell line [7]. In the current study, we conducted a case-control study to investigate the association between this polymorphic *IRGM1* allele and human susceptibility to TB disease.

## Methods

A total of 370 African American and 177 Caucasian adult patients with TB disease without a history of immunosuppressive conditions or diabetes were identified in the Houston Tuberculosis Initiative database (10). The mean age was 47.9±13.2 yrs, and 70.6% were male. Within this group, 469 had pulmonary TB, 59 had extrapulmonary TB, and 26 had both pulmonary and extrapulmonary TB. A total of 180 African Americans and 110 Caucasian adults without history of TB, autoimmune disease, or other infectious diseases were enrolled as controls. All of the subjects were HIV seronegative.

The study was approved by the Institutional Review Boards of the Baylor College of Medicine and The Methodist Hospital Research Institute. After informed consent was obtained, genomic DNA was obtained from peripheral blood samples of study subjects using the Nucleon DNA extraction and purification kit (Amersham International and Scotlab, Buckinghamshire, England). We used SNP *rs10065172* as a marker of the polymorphic allele in this study. SNP *rs10065172* was amplified and sequenced with a primer pair: 5′-TGTCGTACCCAAGCAGAGTG and 3′-GGAGTTTTGCCCACATGTCT and an ABI 3730 XL DNA analyzer using Big Dye Terminator (PE Applied Biosystems). By sequencing with two primers, we obtained information for each sample in duplicate; any incongruous samples were excluded from the final analysis. All raw data were assembled and analyzed by 2 individuals blinded to the analysis (JH and JL). Genotypic frequencies were compared using a chi-squared test for 2-by-2 contingency tables in Excel and EpiInfo software (*P*<0.05 considered statistically significant). The power of the study was calculated to be 37% to detect a 10% difference in allele frequency with 95% confidence, based on the sample size used.

For RNA analysis, peripheral blood mononuclear cells were isolated from peripheral blood by Ficoll separation and preserved in RNA lysis buffer (RNaqueous, Ambion). cDNA was manufactured using random priming and Superscript II reverse transcriptase (Invitrogen). *IRGM1* expression was measured by real-time quantitative PCR using Taqman Assay-on-Demand (Applied Biosystems). Data were analyzed by 1-way ANOVA in GraphPad Prism software.

## Results

**Figure 1:**
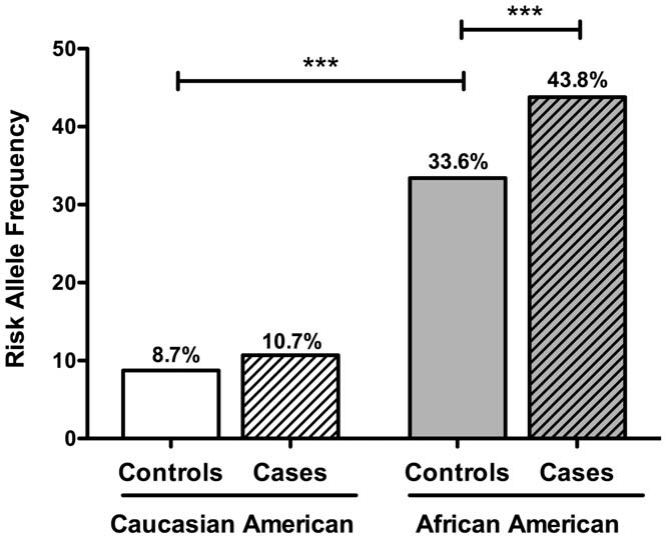
The risk allele frequency for SNP rs10065172 is higher among African Americans with TB disease compared to unaffected controls. For each group the bar and n are as follows: Caucasian controls (white) n = 110; Caucasian cases (white stripes) n = 177; African American controls (grey) n = 180; African American cases (grey stripes) n = 370. Differences (***) are significant (*P*<0.001) by chi-squared test.

The allele frequencies of the polymorphic allele in test groups are shown in Figure 1. Among the Caucasian controls, the allele frequency of *rs10065172* was 8.7%, which is similar to the reported frequencies among Caucasian control groups in previous studies [8]. Among the Caucasian case group, the allele frequency of *rs10065172* was 10.7%, not significantly different from the frequency in the control group with odds ratio (OR) = 1.27, 95% confidence interval (CI) 0.71–2.27 (*P* = 0.48). Among African Americans, the polymorphic allele frequency was 33.4% for controls, consistent with an intermediate frequency between the frequency in a Nigerian population and that of a European Caucasian population according to HapMap (8). However, among African American cases, the allele frequency of *rs10065172* was 43.8%, significantly higher than the control group (OR = 1.56; 95% CI, 1.17–2.02; *P* = 0.001) (Figure 1).

Comparison of genotype frequencies between cases and controls are shown in Table 1. Among African Americans, the number of individuals who are carriers for the polymorphic allele (including both those homozygous and heterozygous for the polymorphic allele) was significantly greater among cases compared to controls, with odds ratio of 2.30 (95% CI, 1.59–3.32) (*P*<0.01). The frequency of the homozygous polymorphic genotype (TT) was not different, but the frequency of the homozygous reference genotype (CC) was lower among African American cases compared to controls, with odds ratio of 0.56 (95% CI, 0.33–0.96) (P<0.05) when expressed relative to the frequency of the TT genotype. There were no significant differences in the genotype frequency between Caucasian case and control groups. The same differences were noted for African American but not Caucasian study groups when the cases of pulmonary TB were analyzed separately from the cases of extrapulmonary TB (data not shown).

**Table 1:** Genotypic comparison of *IRGM1* rs10065172 between tuberculosis cases and controls.

	Caucasians			African Americans		
Genotypes/Allele	CasesN = 177	ControlsN = 110	OR (95%CI)	CasesN = 370	ControlsN = 180	OR (95%CI)
CC	140 (79.2%)	92 (83.5%)	1.0	107 (28.9%)	87 (48.3%)	1.0
TT	1 (0.6%)	1 (1%)	1.52 (0.094–24.6)	61 (16.5%)	28 (15.3%)	1.77 (1.04–3.01)^a^
CT	36 (20.2%)	17 (15.5%)	0.72 (0.38–1.35)	202 (54.6%)	65 (36.3%)	2.53 (1.70–3.76)^b^
CT+TT(carrier)	37 (20.8%)	18 (16.5%)	0.74 (0.40–1.38)	263 (71.1%)	93 (51.7%)	2.30 (1.59–3.32)^b^

Note:

a *P*<0.05.

b *P*<0.01.

In order to investigate the effect of the risk allele on expression levels of IRGM1, we conducted real-time PCR analysis on cDNA samples from peripheral blood mononuclear cells, paired with genotyping of the same cell samples. Among a sample of 76 randomly selected individuals, 5 individuals were homozygous for the risk allele and 19 were heterozygous. There were no differences in overall IRGM1 expression level in these peripheral blood mononuclear cells based on genotype (data not shown).

## Discussion

The genomic sequence including SNPs *rs13361189* and *rs10065172*, as well as a 20-kb deletion/insertion region has been observed at the same genomic location in the chimpanzee genome, indicating its ancestral origin [9]. The 3′ end of 20-kb deletion is about 2.7-kb before the reported *IRGM1* transcription start; and the deletion may affect the function of the *IRGM1* gene promoter, causing decreased gene expression [7]. Our data provide the first evidence of the association between this polymorphic allele of the autophagy-related gene *IRGM1* and human susceptibility to TB disease.

We noted that the frequency of the *IRGM1* polymorphic allele was higher among African American cases compared to controls; conversely the homozygous reference genotype (CC) was underrepresented among African American cases. This finding indicates that the reference allele may be associated with protection again tuberculosis in this population.

Two recent studies have reported an association between polymorphisms in the regulatory region of *IRGM1* and TB [10], [11]. A case-control study conducted in China examined a 1.7-kb promoter region of *IRGM1* and identified one polymorphism, -1208A/G, that was associated with TB susceptibility [10]. Unfortunately, this studied region covered none of the three linked polymorphisms investigated here: *rs13361189*, *rs10065172*, and the 20-kb deletion/insertion. Another study conducted in a West African population found that an *IRGM1* SNP -261T confers protection against a Euro-American lineage of *M. tuberculosis* [11]. However, the CD-related *IRGM1* allele (*rs10065172*) failed to show a significant association with TB disease in that study population [11]. There may be several reasons for the discrepancy between our findings and those of the West African study. For example, the importance of IRGM1 may be dependent on the specific strain of *M. tuberculosis* prevalent in a geographic area. Indeed, specific mutations in the *IRGM1* gene may have differing degrees of impact according to geographic populations, as has been reported in previous association studies [12], [13]. Our study is also limited by the inherent genetic heterogeneity within the African American population; and differences in genetic heterogeneity may exist between our case and control groups. Duplication of this study in another large African American population would strengthen our findings and would be interesting to pursue in the future.

We did not detect a relationship between *IRGM1* expression level and genotype in peripheral blood mononuclear cells. Previous studies have indicated that baseline *IRGM1* expression varies significantly according to cell type. Of note, one study showed that the deletion polymorphism upstream of *IRGM1* led to increased expression in some cell lines (notably, a lymphoblastoid line) while contributing to decreased expression in other cell lines (notably, a colon carcinoma cell line) [8]. A limitation of our study is that the study sample of peripheral blood mononuclear cells contained a heterogeneous mix of cell types that may have obscured any phenotypic changes in *IRGM1* expression. Furthermore, our small sample size would have been underpowered to detect small differences in expression.

The allele frequency of the studied *IRGM1* allele was found more commonly in African and Asian populations (55–60%) compared to Caucasian populations (5%) (http://hapmap.ncbi.nlm.nih.gov). This data is consistent with the findings in our previous TB susceptibility studies, in which TB-related alleles of *NRAMP1, IL8, TLR1, TLR6, TLR10*, and *NOD2* genes are found more commonly in African and Asian-derived populations compared to European-derived populations [12], [13], [14], [15].

In summary, our findings indicate that the polymorphic allele of IRGM1 contributes to susceptibility to TB disease among an African American population within the United States. Furthermore, these findings strengthen a potential link between Crohn's Disease and an infectious etiology through the process of autophagy-dependent immunity. Irgm1 prevents interferon-induced cell death among T cells in mice[16]. Hence, we speculate that IRGM1 similarly participates in regulation of the interferon-dependent immune response in humans, and that dysregulation of the gene leads to misdirected immune responses, manifest as progression to TB disease or Crohn's disease in various populations.
